# Explainable Artificial Intelligence in Paediatric: Challenges for the Future

**DOI:** 10.1002/hsr2.70271

**Published:** 2024-12-12

**Authors:** Ahmed M. Salih, Gloria Menegaz, Thillagavathie Pillay, Elaine M. Boyle

**Affiliations:** ^1^ Department of Population Health Sciences University of Leicester Leicester UK; ^2^ William Harvey Research Institute, NIHR Barts Biomedical Research Centre, Queen Mary University of London London UK; ^3^ Barts Heart Centre, St Bartholomew's Hospital Barts Health NHS Trust, West Smithfield London UK; ^4^ Department of Computer Science Faculty of Science, University of Zakho Zakho Kurdistan Region Iraq; ^5^ Department of Engineering for Innovation Medicine University of Verona Verona Italy; ^6^ Research Institute for Health Related Sciences, University of Wolverhampton Wolverhampton UK

**Keywords:** challenges, explainable artificial intelligence, interpretation, paediatrics

## Abstract

**Background:**

Explainable artificial intelligence (XAI) emerged to improve the transparency of machine learning models and increase understanding of how models make actions and decisions. It helps to present complex models in a more digestible form from a human perspective. However, XAI is still in the development stage and must be used carefully in sensitive domains including paediatrics, where misuse might have adverse consequences.

**Objective:**

This commentary paper discusses concerns and challenges related to implementation and interpretation of XAI methods, with the aim of rising awareness of the main concerns regarding their adoption in paediatrics.

**Methods:**

A comprehensive literature review was undertaken to explore the challenges of adopting XAI in paediatrics.

**Results:**

Although XAI has several favorable outcomes, its implementation in paediatrics is prone to challenges including generalizability, trustworthiness, causality and intervention, and XAI evaluation.

**Conclusion:**

Paediatrics is a very sensitive domain where consequences of misinterpreting AI outcomes might be very significant. XAI should be adopted carefully with focus on evaluating the outcomes primarily by including paediatricians in the loop, enriching the pipeline by injecting domain knowledge promoting a cross‐fertilization perspective aiming at filling the gaps still preventing its adoption.

## 1 Explainable Artificial Intelligence

Artificial intelligence (AI) [1] has shown considerable success in healthcare applications including paediatrics [2]. However, its success and promise has been accompanied by vagueness around how these models arrive at decisions, particularly with models based on deep learning. Explainable artificial intelligence (XAI) emerged to remove the “mystery” around the decision‐making and actions in these models, and to make them more easily accessible and interpretable from a human perspective. Various tools and algorithms have been developed for this purpose. XAI aims to make the decision‐making process transparent and trustworthy, detect biases in model decisions, minimize errors in the model and improve feature engineering [3].

A recent survey [4] gathering a wide spectrum of clinical studies highlighted scepticism regarding the potential of AI in overcoming current barriers to adoption in paediatrics. In particular, 20 articles were selected for data abstraction and analysis, with three consistent themes emerging from these articles, out of which 11 addressed the current state‐of‐the‐art application of AI in diagnosing and predicting health conditions such as behavioral and mental health, cancer, syndromic and metabolic diseases, and four set out future opportunities for AI to be adapted, such as the incorporation of Big Data, cloud computing, precision medicine, and clinical decision support systems. Of note, five considered specific challenges of using AI in paediatric medicine related to data security, handling, authentication, and validation. In addition, another [5] focused on the ethical implications of using paediatrics data in AI algorithms, stressing the need for specific and targeted policies relying on ethical principles and ensuring a fair and secure management of the data.

Overall, although XAI has been employed in different medical applications, its utility in paediatrics [6] is limited compared to that in adult practice. In addition to the issues discussed above, there are multiple complexities [7], including those specifically related to data acquisition, which is the cornerstone of AI and XAI models. Inherent limitations include sample size, external validation, generalizability, explanation level, trustworthiness, causality and intervention, and XAI evaluation. These limitations have a significant impact on AI models, which ultimately affect the outcome of XAI. Therefore, when end‐users in paediatrics interpret the outcome of XAI, these limitations should be carefully considered.

## 2 Potential Challenges in Utility of XAI in Paediatrics

Although XAI has transformed complex models into a more digestible form for human understanding, it is still in the development phase. There are many concerns that necessitate careful use in paediatrics, some of which are directly related to XAI, and others indirectly related through the AI models, data, and the research area. These concerns include trustworthiness, causality, intervention, and the validation of XAI outcomes.

### 2.1 Sample Size

Successful development of AI models relies on having a large sample of data. Small sample sizes can lead to poor model performance and subsequent untrustworthy results. Data acquisition is often more challenging in studies in children compared with those in adults, for a variety of reasons [8]. These include the logistics of conducting paediatric projects with large sample sizes, due in part to costs, less frequent access to healthcare, and heterogeneity in the sample because of the changes associated with ongoing child development and puberty. Large‐scale, routinely collected (real‐world) datasets in paediatrics hold potential for future AI and XAI analyses but require data entry at the point of collection to be as complete as possible, accurate, and validated [9]. One potential solution to overcome the issue of small sample size is to use transfer learning, where learning gained from one task is used to augment functions in another related task. However, most of the trained models (dataset is used to train a machine learning algorithm) use data from adults, which may make them inappropriate for use in paediatrics.

### 2.2 Generalizability

One of the main aims of XAI is to understand the model and to be able to generalize the results across a given population. Generalizability demands that the developed model should perform consistently and reliably on new, unseen data beyond those used for training. This is a special challenge in paediatrics due to the diversity in age, sex, race, ethnicity, indices of social deprivation, child development stages, and sample size [10, 11]. In addition, most paediatric studies train and test a model on a single cohort from a specific population without external validation. The results of such models lack generalizability because there is heterogeneity in phenotypes among the populations. To improve the generalizability of a model, the included data should comprehensively represent the diversity of the expected samples. However, in paediatrics, adequate and complete data collection is challenging for the aforementioned reasons.

### 2.3 Explanation Level

One of the most significant factors in XAI, which has not been considered yet in previous XAI models, is how we would counsel patients, if the material we are utilizing in the counseling, is generated through XAI. XAI, and how the model works should be translatable into a language that patients without technical background can understand [12]. This is somehow more challenging and complex in paediatrics because the counseling needs to be appropriate for situations when dealing with both Gillick competent [13] children, as well as adults.

### 2.4 Trustworthiness

Despite an increasing number of developed AI models in paediatric research, their deployment in clinical practice is still limited. Trustworthiness is one of the biggest barriers to implementing the developed AI model in real‐life clinical cases [14]. End‐users, including paediatric healthcare providers and patients, should be able to trust that the AI model decision is not biased, and that noise and artifacts in the data do not mask clinically significant findings. They need to feel confident that variation related to ethnicity and age has been considered, and that timely detection of any mistake in the system will be possible. In paediatrics, the degree of confidence in the accurate performance of a model may need to be higher, because of the vulnerability of the patient group, with neonates being a clear example for whom even a small error in decision‐making might have substantial adverse effects [15].

In imaging studies, increased motion artifacts can limit both the quality and quantity of usable data, witnessing a harder task than for adults and raising obvious data collection issues. Paediatric studies that have employed AI models have often been limited by the noise especially affecting small data samples.

### 2.5 Causality and Intervention

Early identification of children who are susceptible to poor health outcomes in the future and timely targeted intervention is an important part of paediatric care [16]. Accordingly, when AI models are implemented in such scenarios, the distinction of causality from association should be carefully noted. Current models are reliant on statistical relationships between the input data and the outcome of interest. However, correlation does not imply causation and those models do not represent a causal inference between the input and the output. This means that when XAI identifies specific variables that affect, for example, child wellbeing, it does not necessarily mean that an intervention related to those factors would improve child wellbeing. Raising awareness about this issue is essential for disease prevention and treatment.

### 2.6 Age‐Related Differences in Manifestations of Disease

The prevalence, severity, mortality rate, and the risk factors for the same spectrum of diseases might differ between children and adults. For instance, paediatric asthma is more common in males while in adults, it is predominant in females. The severity of asthma in childhood is determined through factors including impaired lung function, duration of the symptoms, and is associated with ethnicity and socioeconomic deprivation. In adults, the associations are different, including obesity and smoking [17]. This difference in clinical spectrum is observed in other diseases including inflammatory bowel disease [18], celiac disease [19] and cystic kidney diseases [20]. Accordingly, XAI outcomes generated from a model to perform classification of asthma in adults cannot be tested, extended, or generalized to children with asthma. Doing so would generate a false explanation or the model might fail because paediatric data would be considered as having a different distribution for the model when compared to adults. Therefore, simply including a disease, without consideration of the age‐dependant risks and associations does not always mean that the model will understand, perform well, or generate a trustworthy explanation when it is applied to another age group. The model should be trained, tested, and validated using paediatric data so the generated outcome reflects the phenotypical differences due to that pathology at that stage of life.

### 2.7 XAI Evaluation

XAI outcomes need to be validated appropriately, especially when implemented in a sensitive domain where misinterpretation can have important consequences. XAI is affected by several issues related to causality, feature collinearity, susceptibility to adversarial attacks, and generalizability. Criticisms with respect to XAI methods assessment have been acknowledged by pioneering works [21] with respect to sensitivity and implementation independence. This issue is still far from being solved and limits the exploitability and adoption of XAI in critical domains [22]. Despite the many attempts to develop proxies and methods to assess XAI outcomes quantitively, no formal definition or standard measure exists so far to determine whether one specific XAI approach should be considered over others. Another form of evaluation involves considering the views of experts in the field of paediatrics. Although evaluation of XAI based on expert opinion is time‐consuming, subjective, and costly, it may be prudent to involve clinical experts in the progression, further development, and improvement of XAI techniques.

## 3 Future Direction

To increase trust in XAI models in paediatrics, models should be inclusive and consider the specific characteristics and complications relevant to this clinical field. When applying XAI, full information should be reported regarding the data and models used, as well as the age range, ethnicity, and social demographic characteristics of the sample to allow readers to consider whether the current outcomes of XAI are also applicable to other paediatric populations. Specific algorithms and models need to be developed to process infant and child imaging modalities accounting for potential complications related to motion and noise artefacts. Largely because these characteristics and complexities have not yet been fully considered in AI models in paediatrics, evaluating XAI outcomes based only on proxies and statistical measures is not yet a reality in clinical practice. End‐users should be cautious when interpreting the outcome of XAI. However, as XAI is likely to be an inevitable complement to AI in paediatrics further work is needed to ensure that it can be used safely and effectively in the future.

## Author Contributions

**Ahmed M Salih:** conceptualization, writing–original draft. **Gloria Menegaz:** conceptualization, writing–review and editing. **Thillagavathie Pillay:** conceptualization, writing–review and editing. **Elaine M Boyle:** conceptualization, writing–review and editing; supervision.

## Conflicts of Interest

The authors declare no conflicts of interest.

## Transparency Statement

The lead author Ahmed M. Salih affirms that this manuscript is an honest, accurate, and transparent account of the study being reported; that no important aspects of the study have been omitted; and that any discrepancies from the study as planned (and, if relevant, registered) have been explained.

## Data Availability

Data sharing does not apply to this article as no new data were created or analyzed in this study.
